# Characterization of the *Rhipicephalus* (*Boophilus*) *microplus* Sialotranscriptome Profile in Response to *Theileria equi* Infection

**DOI:** 10.3390/pathogens10020167

**Published:** 2021-02-04

**Authors:** Patrícia Paulino, Gabriela Vitari, Antonio Rezende, Joana Couto, Sandra Antunes, Ana Domingos, Maristela Peckle, Carlos Massard, Flávio Araújo, Huarrisson Santos

**Affiliations:** 1 Department of Epidemiology and Public Health, Federal Rural University of Rio de Janeiro (UFRRJ), BR 465, Km 7, Seropedica, RJ 23890000, Brazil; patgpaulino@gmail.com (P.P.); gabriela_vitari@hotmail.com (G.V.); 2 Department of Microbiology, Institute Aggeu Magalhães—Oswaldo Cruz Foundation (FIOCRUZ), Recife, PE 50670-420, Brazil; antonio.rezende@cpqam.fiocruz.br; 3 Global Health and Tropical Medicine, Instituto de Higiene e Medicina Tropical, Universidade Nova de Lisboa, 1349-008 Lisbon, Portugal; jmanuel.couto@ihmt.unl.pt (J.C.); santunes@ihmt.unl.pt (S.A.); adomingos@ihmt.unl.pt (A.D.); 4 Department of Animal Parasitology, Federal Rural University of Rio de Janeiro (UFRRJ), Seropedica, RJ 23890000, Brazil; maristelapeckle@yahoo.com.br (M.P.); carlosmassard@ufrrj.br (C.M.); 5 Rene Rachou Research Center (CPqRR), FIOCRUZ, Belo Horizonte, MG 30190-002, Brazil; araujo@cpqrr.fiocruz.br

**Keywords:** equine piroplasmosis, RNA-seq, tick defense, tick-protozoan interactions

## Abstract

This study intends to characterize the sialotranscriptome profile of *Rhipicephalus (Boophilus) microplus* in response to *Theileria equi* and identify genes of interest with differential genomic expression, indicating relevant targets in the tick–protozoan interactions. The experimental design consisted of RNA sequencing from uninfected and *T. equi*-infected *R. microplus* salivary glands (SGs) to obtain transcriptomic profiles for characterization and comparison. A total of 288,952 transcripts were obtained from both tick profiles, 3456 transcripts (*p* < 0.05) differentially expressed in response to *T. equi* infection. The uninfected SGs’ registered 231,179 transcripts, of which 155,359 were annotated. The most transcribed sequences were female-specific histamine binding protein and lipocalins. Regarding the *T. equi*-infected SGs, from the 238,964 assembled transcripts, 163,564 were annotated. The most transcribed sequences were histone demethylase JARID1 and Y-box-binding protein. Five transcripts (cystatin, arginase, nuclear factor κB kinase inhibitor subunit β (IκB), IκB delta, lysosomal-trafficking regulator, and reeler protein) presented the gene ontology (GO) category “response to protozoan” and were exclusively displayed in the *T. equi*-infected profile. The transcriptome of *T. equi* was also analyzed, registering 4728 hits. The study’s genetic and molecular information would be of great value for future studies and biotechnological applications envisaging disease control.

## 1. Introduction

Equine piroplasmosis (EP) is a tick-borne disease caused by *Theileria equi* and *Babesia caballi*, affecting horses, donkeys, ponies, mules, and zebras, endemic in many parts of the world and posing as an enzootic threat to non-endemic areas [1]. The transmission occurs mainly by vectors, although iatrogenic and transplacental transmission forms have already been reported [2]. According to the World Health Organization, it is a compulsory notification disease when both or only one etiologic agent is identified on the vertebrate host, and the infection can be considered acute, subacute, or chronic [1]. The incubation period is approximately 15 and 20 days for *T. equi* and *B. caballi*, respectively [2]. The characteristic clinical signs are fever, anemia, jaundice, hemoglobinuria, and weakness. Abortions and deaths of neonates are also reported when *T. equi* is present in the infection [2]. This disease can evolve death, but the most common outcome is developing the chronic disease, which, in many cases of *T. equi* infection, persists for long periods, making the equids carriers.

Moreover, the worldwide prevalence of *T. equi* obtained by molecular studies is estimated at 34.6% (95% CI: 34.48–34.76) [3]. Such consequences of the *T. equi* infection directly impact the horse performance and paves the way for a source of infection for competent vectors and healthy animals [4,5]. Besides the impact on animals’ health, this disease substantially impacts the equestrian, commercial world since it is one of the main impediments to horses’ international transit [4].

The protozoan *T. equi* is reported in a considerable number of countries overlapping with the presence of its competent vectors [6]. To date, four distinct genera have been identified to include the experimental vectors of *T. equi*, such as *Dermacentor*, *Rhipicephalus*, *Hyalomma*, and *Haemaphysalis* [6,7]. Thus far in the Americas, only *Dermacentor variabilis* and *Rhipicephalus microplus* were recognized as *T. equi* vectors in experimental conditions [8,9,10]. The *D. variabilis* species is mainly in North America, while *R. microplus* is well distributed in Latin America [6]. Moreover, alongside *Dermacentor nitens* and *Amblyomma sculptum*, *R. microplus* is one of the most present tick species in horses from Brazil [11]. However, its role as the primary vector of *T. equi* is still unclear due to this hypothesis’s *R. microplus*’ contradicting points. As opposing factors, *R. microplus* is a monoxenic ectoparasite, allied to the *Theileria’s* lack of transovarial transmission, reducing the chance of spreading the pathogen. Despite these characteristics, experimental studies have proven *R. microplus* cross-state and intra-state experimental vector competence [8,9].

Furthermore, epidemiological studies in Brazil report an alarming situation concerning the remarkably high *T. equi* prevalence, nearly 90% in some areas, and *R. microplus* is frequently associated with *T. equi* infections around the country [11]. These prevalence rates may also be linked to the treatment inability to eliminate the parasite due to *T. equi* resistance to drugs, resulting in the pathogen’s persistence in the infected vertebrate host for years and possibly throughout the animal’s life [2]. Therefore, ticks control plays a crucial role in the prevention of EP disease. 

The classic ticks’ population control technique is to apply various chemical formulations of ectoparasiticide in the animals [2]. Nevertheless, the excessive use of these chemicals has generated several consequences, such as limited effectiveness in reducing infestation, selection of ticks resistant to the artificial formula, contamination of the environment, as well as meat with drug residues [12]. Among the alternative approaches studied, vaccines that induce vertebrate hosts’ immune protection against tick infestation have gained great research focus [12]. The development of these vaccines may allow the inclusion of multiple antigens that fight ticks and prevent pathogens’ transmission [13]. Reducing pathogens transmission by ticks has become an equally important goal in developing new vaccines. For the effective development of recombinant vaccines, a broad study of the tick–pathogen–host dynamics is necessary [12]. Understanding tick response and pathogen and host interaction are of the utmost importance to discover new potential targets for vaccine candidates in the piroplasmosis prophylaxis.

The salivary glands (SG) are vital anatomical parts of ticks and strategic for parasitic success, considering that pathogen transmission occurs via tick saliva injection into the host [14]. Due to the great importance of this organ, especially in a tick–pathogen interactions, this tissue has been the target of many studies involving gene expression analysis against parasitic infections and characterization of compounds present in salivary secretions [15,16]. Studies have reported that gene expression in a tick SGs is modified in response to infection by pathological agents [15,16]. For instance, *R. microplus* SG’s gene expression in response to *Anaplasma marginale* infection reported 279 differentially expressed genes (*p* < 0.05) [16]. Another example is presented in a recent study that described several changes in the *R. bursa* sialotranscriptomic profile in response to *Babesia ovis* infection [15]. 

By analyzing the sialotranscriptome, numerous proteins involved in the triad *T. equi*-tick–horse interaction can be identified and indicated to develop immunogens to control *T. equi* infection in both the vertebrate host and the invertebrate, as seen in other vector–parasite models [13]. Over the last years, focusing on such interactions has advanced the understanding of the molecular factors connected to the acquisition, survival, and transmission of pathogens [17]. For instance, research in the *Ixodes scapularis* and the *Borrelia burgdorferi* interplay has revealed that the bacteria have a specialized outer surface protein C, which binds the *B. burgdorferi* to a tick salivary protein 15 (Salp15), providing protection from tick’s immune response and permitting the pathogen transmission to vertebrate host [18]. Similarly, the investigation on the piroplasm *Babesia* spp. and *Rhipicephalus* ticks have demonstrated a differential expression of Subolesin (SUB) and its role in the vector–pathogen relationship. Moreover, the SUB participation in the *B*. *bigemina* infection has been supported by a display of decreased pathogen levels in ticks fed on hosts immunized with recombinant SUB [19]. Other *Rhipicephalus* spp. SGs molecules have also been functionally screened for their impacts on *Babesia* infection, such as Lachesin or a Ubiquitin ligase, showing targeting key proteins at the pathogen–SGs interface is a valid approach in the identification of potential immunogens [20]. 

Furthermore, other vector–pathogen investigations have indicated that some tick–parasite interplay may positively contribute to increased tick fitness [17]. For example, another study investigating *I. scapularis* and *B. burgdorferi* shows that the bacteria facilitate tick’s blood-feeding by increasing the blood flow to the tick-bite site and modulating vascular permeability [21]. The balance between the benefits and the harms that this relationship may bring is associated with this co-evolution [22]. However, research aiming to characterize the interactome between *Theileira* spp. and ticks is scarce, and it is worth mentioning that its relationship is clearly understudied. 

Therefore, this study aims to characterize the sialotranscriptome of *R. microplus* during *T. equi* infection and compare the ticks’ uninfected and infected transcriptomic profiles in order to contribute to the advancement of scientific knowledge concerning tick–protozoan interactions and pinpoint molecules that may interfere with pathogen survival and transmission that can be focused on future studies. 

## 2. Results

Infection of SGs in the ticks allowed to feed on the *T. equi* infected horse was confirmed before experimentation, and quality checked total RNA was used in RNA-Seq analyses.

### 2.1. Raw RNA-seq Data

The RNA-seq quality was considered satisfactory by the FastQ quality check algorism, and most of the sequences were between points 30 and 40 on the Phred scale, which ranges from 2 to 40 points. Additionally, the absence of “N” content in the reads indicates good sequencing quality. As expected, the average length of the reads was 150 bp. 

### 2.2. Transcriptome de novo Assembly 

The de novo sequence assembly resulted in 295,406 putative transcripts with a percent GC-content of 46.21% and a mean length of 340 nucleotides (nt). According to the standard quality metrics for the transcriptomic assemblies (ExN50), 50% of the assembled bases were incorporated in transcripts of 2721 nt in length. The detailed assembly statistic is described in Table 1. However, 6471 sequences (2.19%) were discarded due to their small size. From the overall 288,952 transcripts, the uninfected samples presented 231,179, in which 49,988 were exclusive to this group, while the *T. equi*-infected samples presented 238,964, in which 57,773 were exclusive to this group.

The predicted coding region analysis resulted in 99,621 transcripts, of which 62,987 had a completed open reading frame (ORF), which is the part of a nucleotide sequence that has the potential to be translated, 18,106 had a 5’ partial ORF, 8026 had a 3’ partial ORF, and 10,502 had an internal ORF. The potential coding analysis presented a potential coding cutoff of 0.701 and a non-coding potential cutoff of 0.224 (accuracy of 0.95).

The assembled transcriptomes were assessed for completeness using Benchmarking Universal Single-Copy Orthologs (BUSCO) analysis. The arthropod-related groups reported 94.47% complete gene representation (single-copy or duplicated), while 0.99% was only partially recovered, and 4.54% were missing. The Apicomplexa-related groups reported 95.74% complete gene representation (single-copy or duplicated), while 0.45% were only partially recovered, and 3.81% were missing.

### 2.3. Annotation of Sialotranscriptome Profiles

Annotation of the uninfected samples’ assembled sequences resulted in 67.20% (155,359/231,179) hits successfully identified by the Basic Local Alignment Search Tool (BLAST) algorithm from the National Center for Biotechnology Information (NCBI) database (Supplementary Table S1). From these, 155,152 were annotated as arthropod related, 146 were identified as the equine host, and 61 were associated with endosymbionts (*Coxiella* sp., *Rickettsia* sp., and *Francisella* sp.). An amount of 14,838 transcripts were identified exclusively in the uninfected samples. In the case of the assembled sialotranscriptome of the *T. equi*-infected samples, 68.45% (163,564/238,964) sequences were successfully identified by the BLAST algorithm, where 158,600 were associated with arthropods, 130 with the equine host, and 264 with endosymbionts (*Coxiella* sp., *Rickettsia* sp., and *Francisella* sp.) (Supplementary Table S1). An amount of 23,043 putative transcripts were identified exclusively in the *T. equi*-infected samples. The presence of *Theileria* in the SGs allowed identifying 4728 putative transcripts belonging to the protozoan (Supplementary Table S1). 

### 2.4. Functional Analysis of Sialotranscriptome Profiles

#### 2.4.1. *Rhipicephalus microplus* Sialotranscriptome Profiles

Gene ontology (GO) is a standardized annotation of gene products applied to identify characteristic biological attributes of genomic data. The assembled transcripts’ functional analysis resulted in 98,214 GO annotations; 18,004 records were found exclusively in the *T. equi*-infected samples and 11,301 exclusively in the uninfected samples (Supplementary Table S1). 

**Figure 1:**
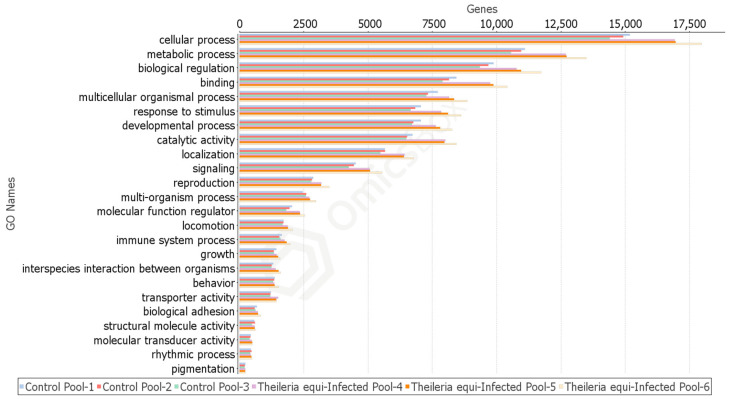
Graphic representation of gene number distribution on each gene ontology (GO) category according to the *Rhipicephalus microplus* transcriptome profiles.

Cellular and metabolic processes, biological regulation, binding activity, multicellular organismal process, response to stimulus, developmental process, catalytic activity, localization, and signaling are the top classifications. These categories present a remarkable difference in representatives’ number, favoring the *T. equi*-infected profile (Figure 1). Categories such as multi-organism process, molecular function regulator, locomotion, immune system process, and interspecies interaction between organisms also display a significant difference in representatives’ number favoring the *T. equi*-infection group. There is no apparent difference in behavior, structural molecule activity, molecular transducer activity, rhythmic process, and pigmentation sections.

Furthermore, the GO profile comparison revealed that the transcripts: cystatin, arginase, nuclear factor κB kinase inhibitor subunit β (IκB), IκB Delta, lysosomal-trafficking regulator, and reeler protein were directly related to tick’s defense against the pathogen since these sequences were tagged by GO analysis with the "response to protozoan" classification, and this category was presented exclusively in the *T. equi*-infected profile.

#### 2.4.2. *Theileria equi* Transcriptomic Profile during Sporogony

From a total of 4728 BLAST-identified transcripts, 58.43% (2763/4728) presented a GO classification. The Interpro analysis has provided 492 additional classifications. 

Direct count of GO classifications shows that the most prevalent biological process is the oxidation–reduction process, transmembrane transport, and xenobiotic transport. The most prevalent molecular functions were adenosine triphosphate (ATP) binding, ATPase activity, 2-alkenal reductase activity, and ATPase-coupled xenobiotic transmembrane transporter activity. The most prevalent cellular components are an integral component of the membrane and apical plasma membrane.

Interpro analysis indicated that the most prevalent matches were a protein of unknown function DUF529 [PF04385], followed by serine aminopeptidase, S33 [PF12146], RAP (an acronym for RNA-binding domain abundant in Apicomplexans) domain [PF08373], membrane attack complex component/perforin (MACPF) domain [PF01823], and merozoite antigen [PF02488].

#### 2.4.3. GO Terms in *Theileria equi* Profile Highlights Parasite–Host Interactions

The *T. equi* transcriptome profile presented transcripts with GO annotations indicating involvements in the endocytosis process, such as adenosine diphosphate (ADP)-ribosylation factor, which is recognized as a protein localized in the phagophore assembly site. Multiple ATP-binding cassette (ABC) transporters are spotted as proteins participating in the parasites’ internalization by clathrin-coated vesicle membrane, while others engage in the early endosome phase, and most are tagged by their location on the apical plasma membrane. Other GO terms were registered in the. *T. equi* profile, such as the function of microtubule binding and the attachment of spindle microtubules to the kinetochore. 

Additionally noteworthy is the identification of transcripts involved in “complement activation”, “cellular oxidant detoxification”, and “antioxidant activity". Moreover, a transcript identified as “Hydrolase_4 domain-containing protein” was marked by GO as a transcript involved in the generation of pathogen’ pathogenesis, and it is located in the extracellular region.

Other *T. equi’s* transcripts were found to have the potential to alter the host signaling pathways, for instance, cyclin, which regulates the cyclin-dependent protein serine/threonine kinase activity in the host cell nucleus, a serine/threonine phosphatase (Enzyme code: 3.1.3.16), which may influence the calcium signaling pathway; other transcripts were marked with the function IκB activity, such as cactin protein, which can manipulate the NF-κB cascade. 

*Theileria* spp. also secretes several proteins to modulate the genomic expression of the host cell. An exciting finding was the *T. equi* expression of the AT-hook motif-containing protein. This protein possesses domains required for binding to AT-rich DNA (AT hooks) and transport into the host cell nucleus [23]. 

### 2.5. Gene Expression in Response to Theileria equi Infection

The most expressed transcripts in the control profile were female histamine binding proteins and lipocalins. By contrast, the most expressed transcripts in the *T. equi*-infected profile were histone demethylase Jumonji AT-rich Interactive Domain 1B (JARID1) and Y-box-binding protein.

The total number of differentially expressed genes (*p* < 0.05) obtained was 5257. After selecting tick-only genes, the number of differentially expressed genes (*p* < 0.05) was 3456. The genomic expression was assessed in logarithm fold change (Log FC) measurements, which describes how much a gene expression level changes between the two experimental conditions. A total of 836 transcripts were down-regulated in the infected condition (LogFC < −1), and 2597 transcripts were up-regulated in the infected condition (LogFC > 1). The transcripts with differential expression are enlisted in the Supplementary Tables S2 (down-regulated) and S3 (up-regulated).

#### 2.5.1. Redox Metabolism 

Redox pathways resulting in the production of reactive oxygen species (ROS) and reactive nitrogen species (RNS) are known to be involved in the control of pathogens [24]. Genes encoding ROS-producing enzymes such as Dihydronicotinamide-adenine dinucleotide phosphate (NADPH) oxidase (LogFC = 9.4), sulfhydryl oxidase (LogFC = 10.5), cytochrome C oxidase (LogFC = 1.8), and cytochrome P450 (LogFC = 10) are involved in the oxidative stress response and were here identified as up-regulated in *R. microplus T. equi* infected SGs. Moreover, as a response to the high level of ROS, a high/intense antioxidant activity can be observed in the GO analyses (molecular function), and many genes such as peroxidase (LogFC = 10), peroxiredoxins (LogFC = 9.4), thioredoxin (LogFC = 11), and glutathione-S-transferase (LogFC = 9.3) were found to be up-regulated in the *T. equi* presence. Furthermore, the endosymbiont *Coxiella* sp., identified in the samples, presents various genes that participate in removing superoxide radicals and antioxidant activity, such as glutaredoxin (LogFC = 10.6), superoxide dismutase (LogFC = 10.5), and thiol peroxidase (LogFC = 9.5), respectively, and were found to be up-regulated in the protozoan-infected profile.

#### 2.5.2. Carbohydrate-Related Proteins 

Numerous carbohydrates-related proteins involved in the parasite–host interactions such as cell surface glycoproteins (LogFC = −7.6), the lectins ixoderin B (LogFC = −10), and fibrinogen related proteins (LogFC = −8.8) were down-regulated in the *T. equi* presence.

#### 2.5.3. Antimicrobial Activity

Cystatins were down-regulated in the protozoan-infected group (LogFC = −7.5). Antimicrobial peptides such as defensins (LogFC = −4.5) and ixostatin (LogFC = −6.5) were also down-regulated in the presence of *T. equi*. In contrast, complement proteins such as α-2-macroglobulin (LogFC = 3.3) and complement factor B (LogFC = 3.1) were found to be up-regulated in the protozoan-infected profile. 

#### 2.5.4. Signaling Pathways 

The NF-κB (nuclear factor kappa-light-chain-enhancer of activated B cells) signaling pathway representative tumor necrosis factor (TNF) receptor-associated factor 6 (TRAF6) was down-regulated (LogFC = −6.64), and NF-κB inhibitor-interacting Ras-like protein (NFKIRAS) was identified as up-regulated (LogFC = 6.32) during *T. equi* infection. The adaptor pellino, which represents the Toll signaling pathway, was up-regulated (LogFC = 5.78) in the *T. equi* infection. 

Additionally, calmodulin, one of the most influential proteins in the calcium-mediated signaling pathway, was exclusively present in the up-regulated gene groups with several isoforms. Similarly, mitogen-activated protein kinase (MAPK) cascades were also overrepresented exclusively in the protozoan infection profile with up-regulation of mitogen related proteins and cyclin-dependent kinases. 

#### 2.5.5. Blood Feeding/Mitigation of Host Defenses

The expression of anti-hemostatic proteins (18.3 kDa proteins (LogFC = −4.37), tissue factor pathway inhibitor (LogFC = −9.31), carboxypeptidase inhibitor (LogFC = −5.66), basic tail secreted proteins (LogFC = −11.61) was down-regulated, and cytosolic prostaglandin-E synthase (vasodilator) was up-regulated (LogFC = 11.52) in *T. equi* infection.

Proteins related to evasion of host defenses (DA-P36 proteins (LogFC = −8.61), complement C5 inhibitor (LogFC = −6.210, high-affinity histamine-binding proteins (LogFC = −13.41), and evasins (LogFC = −11.62)), appeared as down-regulated transcripts in *T. equi* infection. 

### 2.6. Transcriptome Validation through qPCR

Thirty genes were selected for the validation of RNA-seq results. The Spearman coefficient (ρ) revealed a strong and significative correlation between RNA-seq and qPCR data (ρ = 0.71/1) (*p*-value < 0.0001). 

## 3. Discussion

*Theileria* parasites are described as one of the “smartest” apicomplexan parasites due to their skill to manipulate the host cells aiding in parasite survival, establishment, and proliferation within the hosts [25]. However, the molecular mechanisms used to achieve such modulation are still unclear [26]. To the best of the authors’ knowledge, this is the first study that focuses on *R. microplus* SG reaction to *T. equi* infection, comprising valuable information that can be further explored towards a more in-depth understanding of the vector–*Theileria* association. Despite not being a study focused on profiling the *Theileria* gene expression, the applied approach allowed an overview into the *T. equi* transcriptome during sporogony, adding to the insufficient knowledge regarding the *Theileria* dynamics inside the tick vector. Thus, the present study builds knowledge on SG cells’ impact and reaction to *Theileria* infection and the parasite modus operandi once reaching the SG tissue. 

### 3.1. Theileria equi’ Invasion Process

As an obligate intracellular protozoan parasite, it gains host cell access by endocytosis through a zipper-like mechanism [26]. Multiple ATP-binding cassette (ABC) transporters expressed by *T. equi* are functionally marked as “clathrin-coated vesicle membrane”, apical membrane, and participating in the early/late endosome stages. This finding may suggest that the endocytosis mechanism is also used by *T. equi* to invade the tick SG cells. 

The Ca^2+^ mobilization is essential for the success of *T. parva* sporozoite’s internalization in bovine lymphocytes [27]. In the *T. equi* transcriptome obtained here, numerous proteins were indicated to play a role in calcium signaling, including calmodulin, an intracellular target for Ca^2+^ activation that acts on proteins such as guanylyl cyclase, protein kinases, and phosphatases to aid in signal transduction, substantiating the involvement of Ca^2+^ in the cell invasion process also in the vector. [28]. 

Parasite engulfment involves recognition by cell-surface receptors to guide the membrane around the target pathogen [26]. The current study reports the adenosine diphosphate ADP-ribosylation factor expression, which is tagged as participating in the endocytosis process because of its location on the phagophore assembly site. Additionally, other transcripts were marked as involved in endocytosis, engaging in early/late endosome phages, or endosome recycling. Another transcript, annotated as hydrolase 4 domain-containing protein with extracellular localization, is defined as a virulent protein (GO category: pathogenesis). This hydrolase may confer the parasite the ability to invade host cells. Alongside other membrane proteins, this protein may interact with the host cell surface to form a tight continuous junction.

### 3.2. An Insight into Theileria Intracellular Maintenance

In contrast to *Plasmodium* spp. and *Toxoplasma gondi, Theileria* parasites dissolve the parasitophorous vacuole’s membrane (PV) after entry into the bovine lymphocytes [29]. Genes encoding for rhoptries and microneme’s proteins were highly expressed in the present study, suggesting that *T. equi* may use the same invasion strategies during the vertebrate host cell invasion. Several transcripts encoding for proteins with functions related to microtube binding and the attachment of spindle microtubules to the kinetochore, such as the 104 kDa microneme/rhoptry antigen (with a primary role in dissolving the enveloping PV membrane), were identified in the transcriptome of *T. equi* located in the SG tissue.

Morphological studies have demonstrated that secretory organelles are not major intervenient in the *Theileria* internalization process but are essential for the parasite’s post-entry establishment [30]. 

After the protozoan entrance in vertebrate cells, *Theileria* resides free in the cytosol closely attached to host cell microtubules [29]. The same parasitic strategy may be taken in the invertebrate host since several proteins with functions related to microtube binding and the attachment of spindle microtubules to kinetochore were found in the *T. equi* profile. The expression of these transcripts may contribute to the parasite cell localization in tick SG cells. 

### 3.3. Theileria equi: Drivers of Cell Changes 

There are some advantages related to the *Theileria* cytosol free-living status, for instance, avoid lysosomal destruction and close contact to modulate host genomic expression [26]. A considerable number of epigenetic factors secreted by *Theileria* are described to instigate vertebrate host cell transformation, such as subtelomeric variable secreted proteins (SVSPs), Tash/TpHN, Tpr/Tar, and TP9/TA9 gene families [31,32,33,34,35]. In the current study, numerous representatives of the Tash family group were identified. These proteins contain the AT-hook DNA-binding motif, which interacts with the cell host and participates in the transformation process. The potential to modulate bovine gene expression of this polypeptide has been previously described; thus, it may have a similar role in the tick salivary gland. 

*Theileria parva* and *Theileria annulata* are known to “trick” their vertebrate host cells into a cancerous phenotype, whereas *T. equi’s* schizont does not appear to incite uncontrolled host cell proliferation [36]. However, the present study observed an opposite situation in ticks’ cells infected with *T. equi*, considering the most expressed transcript encodes a histone demethylase JARID1 and Y-box-binding protein. The JARID1 possesses H3K4 methylation activity, and it is associated with tumorigenicity [37]. Other expressed transcripts encrypting histone methyltransferases are involved in tumor proliferation, particularly histone-lysine N-methyltransferase (SMYD3). *Theileria* infection induces the reversible expression of the histone methyltransferase SMYD3, which also has H3K4 methylation activity [38]. SMYD3 binds specific DNA sequences and activates the transcription of target genes such as matrix metalloprotease-9 (MMP), a critical protein for maintaining cancer status [37]. Therefore, *T. equi* induction of histone methylation in tick SG cells may be one of the alternatives that the parasite uses to hijack the host epigenome. Finding these transcripts in the present study can suggest that some mechanisms can be conserved, but more studies need to be conducted to shed some light on the encoded proteins’ role at the SG interface.

By contrast, the most expressed transcripts in the *R. microplus* free of pathogens sialotranscriptome profile are female-specific histamine binding proteins and lipocalins isoforms linked to the mitigation of host defenses. 

### 3.4. Theileria Endeavors Manipulation of Host Signaling Routes and the Ticks’ Response 

Intracellular parasites are known to hijack the host cell signaling [26], and *Theileria* interferences in host signaling are described in vertebrate host cells [26]. Calcium impacts nearly every aspect of cellular life, binding to numerous proteins to effect changes in localization, association, and function [39]. In the present study, several isoforms encrypting calmodulin proteins were identified in the *T. equi*-infected salivary glands profile. These ubiquitous adaptor proteins are hallmarks of the Ca^2+^ signaling pathway, and their presence suggests an overall high metabolic activity. A past study demonstrates that *Theileria parva* sporozoites require the mobilization of Ca^2+^ for successful parasite internalization [27], interfering this way in the intrinsic balance of cellular Ca^2+^.

A great example of pathogen manipulation is the case of the NF-kB (nuclear factor kappa-light-chain-enhancer of activated B cells) signaling route. The present study shows that *Theileria* expresses functional transcripts encoding for proteins with IκB (inhibitor of kappa-B kinase) activity. These parasite proteins, such as cactin, may mimic the host IκB kinase (IKK) complex phosphorylation of IκB and release the NF-κB complex to enter the host nucleus and activate the cell survival gene’s transcription [40]. In response to *T. equi* infection, *R. microplus* expresses two transcripts marked as “response to protozoan”, the classical IκB, and an atypical IκB called IκB delta, which may neutralize the parasite modulation. This atypical NF-κB regulator interacts with the p65 portion of the NF-κB complex when this transcription factor is inside the nucleus [41]. IκB delta binds p65 only in the nucleus. However, the p50 portion can be bound both in the cytoplasm and in the nucleus [41].

Moreover, *T. equi* infected *R. microplus* also presents three differentially expressed transcripts important to the NF-κB pathway regulation: upregulation of NF-κB inhibitor-interacting Ras-like (NFKIRAS) and downregulation of TNF receptor-associated factor 6 (TRAF6) protein. The NFKIRAS is another atypical protein that acts as a potent regulator of NF-κB activity by rendering IκB more resistant to degradation [42]. Furthermore, the TRAF6 acts as a signal transducer in the NF-κB signaling route that activates the IKK complex in response to a stimulus. The suppression of TRAF6 gene levels may restrict this signaling cascade’s activation, limiting the activation of cell survival genes. Besides the suppression of TRAF6, the adaptor pellino is overexpressed in the *T. equi* presence. Pellino’s is a negative regulator factor of NF-κB/Toll signaling pathways since its function is to mark the adaptor MyD88 (myeloid differentiation primary response 88) for polyubiquitination and degradation [43]. The MyD88 protein is necessary to recruit a cytosolic adaptor tube to the cell surface, thus contributing to NF-κB/Toll signaling transduction [43]. 

Comparing infected and uninfected transcriptomic profiles also shows that the mitogen-activated protein kinase (MAPK) cascade is more active during infection since multiple representants of this signaling pathway, such as mitogen-related and cyclin-dependent kinases, are up-regulated. This pathway is responsible for controlling the cell life cycle, which the parasite may manipulate to increase cell survival [26]. 

One remarkable anti-apoptotic strategy used by this pathogen is the inactivation of the tumor suppressor protein 53 (p53) pathway by abducting p53 protein from the host cytosol to the schizont surface [44]. In *R. microplus*-infected cells, *Theileria* intervention in the p53 pathway is not explicit. However, the *R microplus’* gene TP53-regulated inhibitor of apoptosis 1 (TRIAP-1) was found up-regulated, fold change > 9, in the *T. equi*-infected profile versus the uninfected profile, and some transcripts in the *T. equi* profile were marked with the function of p53 binding. 

### 3.5. Rhipicephalus Microplus Response to Theileria equi: Co-Evolutional Status

The current study presents a high number of transcripts with differential gene expression in the presence of *T. equi* infection. Preceding studies focusing on tick–pathogen relationships have shown that infection does not induce a robust transcriptional response with a relatively low number of differentially expressed genes. In fact, the number of differentially expressed genes of *R. microplus* in response to *A. marginale* was found to be reduced to 279 (*p* < 0.05) [16], and in the case of the *R. annulatus* sialotranscriptome response to *B. bigemina*, only 360 differentially expressed genes (*p* < 0.05) were reported [45]. This fact may be due to these systems’ co-evolutional status since these ticks are recognized as the corresponding pathogen’s natural vectors [22]. The present study, on the other hand, has shown that *T. equi* presence on *R. microplus* SGs resulted in the differential expression of a high number of genes (3456 transcripts (*p* < 0.05)), which may support the concept of a shorter co-evolution. 

Another factor that adds to the current co-evolutional status between *R. microplus* and *T. equi* is the multiple representants of stress-related protein such as heat shock proteins and glutathione-S-transferase with differential expression in the present report and the imbalance of some essential biological activities such as attachment, blood-feeding, and mitigation of host defenses [22]. Ticks typically express a variety of anti-hemostatic proteins and immunomodulators to improve blood-feeding. However, these components seem to be suppressed during *T.* equi infection. The parasite may silence tick genomic expression to boost its transmission by suppressing anti-clotting proteins, such as 18.3 kDa proteins, reprolysins, and tissue factor pathway inhibitor, as well as inducing the expression of the pro-vasodilator cytosolic prostaglandin-E synthetase. 

Interestingly, diverse transcripts encodings proteins related to the evasion of host defenses such as DA-P36 proteins, complement C5 inhibitor, high-affinity histamine-binding proteins, and evasins appeared downregulated during *T. equi* infection. A possible explanation is that equine piroplasmosis leads to immunosuppression of the infected host, thus requiring less expression of immunomodulators from the tick. In addition, as a protozoan infecting blood cells, the suppression of these immunomodulators increases the bite site’s local inflammation process, which may benefit the pathogen, increasing the number of cells subject to infection.

### 3.6. Recognition of Theileria and Antimicrobial Activity 

Diverse cellular functions have been tested in *Theileria* infected cells [26]. The retrieved results suggest that the pathogen recognition activity may be compromised during *T. equi* infection, considering that the *ixoderin B* gene was found to be down-regulated in the infected profile. Ixoderin B is a fibrinogen-related lectin expressed in the SG, and its function is related to the recognition of pathogen-associated molecular patterns (PAMPs) of invading microbes [46]. Another protein that participates in the PAMPs recognition is cystatin, which presents antimicrobial activity as well. The transcript encoding for this protease was also found as down-regulated in the *T. equi*-infected profile, unlike studies made with *Babesia gibsoni* and *Haemaphysalis* ticks [47]. In the *R. microplus* infected profile, cystatin can be found in the GO category of "response to the protozoan", and it is described as having an activity similar to lysosomal cysteine proteinases (cathepsins B, H, L, and S), which participates in the degradation of parasites tissues and the melanization process [47]. Besides cystatin, transcripts encoding antimicrobial peptides such as defensins and ixostatin were also found suppressed in the protozoan-infected group. 

By contrast, two transcripts encrypting thioester proteins such as α-2-macroglobulin (A2M) and complement factor B were up-regulated in the *T. equi*-infected profile. These peptides may perform parasite opsonization or direct lysis, while A2M can also inactivate and clear protease virulence factors [48]. Therefore, these proteins may mark the pathogen for phagocytosis. Moreover, the lysosomal trafficking regulator protein expression is also present in response to *T. equi* infection. This protein is included in the GO category of “endolysosome assembly”/“phagolysosome assembly”, which means the fusion of the late endosome or phagosome to the lysosome. This mechanism enables protozoan destruction by the host lysozymes attack. 

Transcripts encoding for a reeler protein were also present in the *R. microplus* infected profile (GO category: response to protozoan). Proteins containing a reeler domain were described in a manifold of studies involved in the tick’s innate immunity. Previous investigations indicate the participation of this protein in the *Bombyx mori* melanization cascade, and it is also reported as a defense response against the viruses in whiteflies of the *Bemisia tabaci* species complex [49,50]. Apart from insects, there is a study reporting protein with a reeler domain favoring the colonization of *Borrelia burgdorferi* in gut cells of *Ixodes scapularis* [51]. In the current study, it is unclear how this protein-containing reeler domain interacts in the *R. microplus* response to *T. equi*. Further functional and proteomic characterization is needed to clarify this. 

### 3.7. Ticks’ Oxidative Response to Theileria equi Infection and Its Defensive Reaction

Infection by intracellular microorganisms generates an oxidative stress response in vertebrate and invertebrate hosts [2,24]. The production of reactive oxygen species (ROS) by mitochondria is crucial in this response. *Rhipicephalus microplus* tick cells respond to microbial stimuli with ROS production, although some pathogens such as *A. marginale* counteract repressing ROS production and inducing antioxidant genes [52]. The oxidative response in the vertebrate host to *T. equi* infection has already been described, and the horses react with ROS production [53]. The present study shows a similar pattern concerning the response of *R. microplus* to *T. equi* infection—displaying highly up-regulated pro-ROS production genes as well as various antioxidant genes, such as thioredoxins and peroxiredoxins.

As a consequence of the high ROS levels in the infected cells, the *T. equi*-infected profile also displays many isoforms of antioxidant genes, such as superoxide dismutase, transcribed by endosymbiont organism. Symbionts may confer multiples and significant advantages to their hosts [54]. In this study context, the tick’s endosymbionts try to help *R. microplus* regain cell redox homeostasis. However, its efforts may help the parasite to survive, reducing ROS levels. 

A noteworthy finding resides in the fact that, aside from the ROS defense response, the arginase enzyme appears functionally annotated, exclusively in the *T. equi*-infected profile, in the GO category "response to protozoan". Arginase is described to interfere with the nitric oxide (NO) response to pathogens [52]. It competes with NO synthase for its common substrate, L-arginase, affecting NO production. Preceding studies have reported that the *Theileria* protozoan induces the expression of NO inhibitors, which, allied with the finding of this enzyme in the transcriptome of infected SG, supports the existence of a dynamic cellular balance of NO during *T. equi* infection [55]. The exclusive presence of these transcripts in the *T. equi*-infected profiles may illustrate some mechanisms by which the protozoan actives to evade the tick immune response. Controlling such response to pathogens appears to be crucial to favor the host or the pathogen. 

Regarding the protozoan response to the host cell’s high levels of oxidative stress, the *T. equi*’s transcriptomic profile analyses showed the expression of 2-alkenal reductase (EC:1.3.1.74). Despite scarce information, a previous study has reported this enzyme in the genome of *L. amazonensis* [56], and an experiment performed in plants suggested that this enzyme may have a defensive role against oxidative stress by catalyzing the reduction of reactive carbonyls [57].

## 4. Material and Methods

### 4.1. Study Design and Rationale

The study was designed in four stages: (1) first experiment conducted to obtain the biological samples destined to RNA-seq investigation; (2) RNA-seq bioinformatics analysis performed to interpret the collected data; (3) second experimental conducted to obtain biological samples destined to real-time PCR investigation; (4) genomic expression analysis by real-time PCR performed in order to validate the results gathered from RNA-seq technology.

### 4.2. Rhipicephalus microplus Colony

*Rhipicephalus microplus* lineage free of hemoparasites was acquired from the Biological Control Laboratory of Universidade Federal Rural do Rio de Janeiro, supervised by Professor Vânia Bittencourt. Engorged *R. microplus* females from artificially infested animals were extracted from cattle and identified according to the dichotomous key previously described [58]. Ticks were washed with distilled water, dried on paper towels, weighed on a precision scale, packed in a petri dish, and kept in biochemical oxygen demand (BOD) chamber at a temperature of 27 ℃ (±1) and humidity relative higher than 80% until oviposition. After oviposition, each female and a sample of eggs were tested by conventional PCR for pathogen detection (*Babesia* spp., *Anaplasma* spp., *Ehrlichia* spp.) using primers and conditions previously described [59,60]. Eggs from the first three days of oviposition were separated and transferred to adapted sterile disposable syringes, incubated in a BOD chamber under the same conditions described above until hatching of the larvae. After hatching, the larvae were kept in the BOD chamber for two additional weeks for mouthpiece maturation.

#### 4.2.1. Production of Uninfected and Theileria equi Infected Rhipicephalus microplus Ticks

A pilot experiment was conducted to verify which cycle day would present the highest *T. equi* infection levels. During the 21st to the 35th days of the ticks’ life cycle, engorged female specimens were manually removed from the equine host and dissected every day. The *T. equi* infection was detected and quantified by a standard qPCR assay [61]. The 30th day presented the highest number of parasites.

A pathogen-free horse and a horse naturally infected with *T. equi* were used. Diagnostic confirmation was made by cytology and PCR [61]. The animals were kept in individual stalls free of ticks and insects, submitted to sanitary management, and fed with grasses and concentrated commercial rations and water ad libitum. During the experimental infestation, horses’ blood tests were performed once a week to determine horses’ clinical assessment and parasitological quantification. Larvae were applied to each equine host on day zero. During the first three days, the larvae moved around the host bodies to find a suitable fixation place. Thirty days post-attachment, the fully engorged ticks were removed from the host and promptly processed.

#### 4.2.2. Tick Dissection

Following removal, the specimens were immersed in running water, posteriorly in 0.05% sodium hypochlorite solution for three min, rinsed in 75% (*v/v*) ethanol, and finally distilled water. The ticks were dried and dissected under a stereoscopic microscope at 4× magnification (Nikon SMZ745) using sterile conditions in ice-cold solution NaCl 0,9% (Eurofarma, Sao Paulo, Brazil). Thirty salivary glands (SG) were pooled in 2 mL tubes and stored in 1 mL of RNAlater (Thermo Fisher Scientific, Wilmington, DE, USA), resulting in three biological replicates for both control and infected samples.

### 4.3. RNA Extraction, Quantification, and Parasitic Status Confirmation

The SGs were washed with 1 mL of phosphate saline buffer (PSB) and centrifuged for 15 min. This process was repeated three times to remove the RNA later reagent completely. RNA extraction was performed using and according to the RNeasy Mini Kit (Qiagen, Hilden, Germany). Afterward, the samples were submitted to treatment with DNAase I (Thermo Fisher Scientific, Wilmington, DE, USA). An electrophoresis run was performed to verify the samples’ extraction quality and any DNA contamination. The RNA quality was assessed by a Bioanalyzer Agilent RNA 6000 (Agilent Technologies, Santa Clara, California, USA) and quantified via the fluorometric method using commercial kit RNA HS Assay in Qubit 4.0 equipment (Thermo Fisher Scientific, Wilmington, DE, USA).

An aliquot of each condition was submitted to cDNA synthesis to confirm through quantitative PCR (qPCR) detection of *T. equi* in the experimental group and the parasite’s absence in the control group. The detection of *T*. *equi* DNA in the samples employed a TaqMan PCR system in a Real-Time StepOne Plus^®^ instrument to amplify the 81-bp fragment of the *18S rRNA* gene [61]. The assay used the Be18SF (5′-GCGGTGTTTCGGTGATTCATA-3′) and Be18SR (5′-TGATAGGTCAGAAACTTGAATGATACATC-3′) primer set and a fluorescent hydrolysis probe, Be18SP (5′-AAATTAGCGAATCGCATGGCTT-3′), which was labeled at the 5’ end with the reporter dye 6-carboxyfluorescein and the 3’ end with the quencher dye 6-carboxy-tetramethylrhodamine [61]. The reactions were performed in triplicate with a final volume of 12 μL, which contained: 1X TaqMan^®^ Universal PCR Master Mix, 450 nM of each primer, the 250 nM of hydrolysis probe, and 180 ng of total cDNA.

The thermocycling conditions were 50 °C for 2 min, 95 °C for 10 min, and 45 cycles at 95 °C for 20 s, followed by 55 °C for 1 min. Samples with quantification cycle (*C*_q_) values less than or equal to 40 cycles were considered positive. The applied qPCR assay characteristics were established in a prior study, which reports a detection limit of three copies of the *18S rRNA* gene, determination coefficient of 99%, and efficiency of 97.65% [11].

### 4.4. RNA-seq

The total RNA samples were fragmented, followed by purification and cDNA synthesis, using the Truseq Strand mRNA sample preparation kit (Illumina Inc., San Diego, CA, USA), as instructed by the manufacturer. The libraries were quantified using qPCR with the Kappa Sybr Fast qPCR kit (Thermo Fisher Scientific, Wilmington, DE, USA). The samples were diluted in a Tris-HCl solution and 0.1% Tween, deposited in a cartridge, and run on the NextSeq ™ 500 High Output Kit (300 cycles).

### 4.5. RNA-seq Data Analysis

The quality of the raw data was verified by the FastQC 0.11.8 tool. The transcriptome reconstruction was performed in the Trinity platform, and it was based on de novo methodology due to the absence of the *R. microplus* genome available in the online databases. The raw fastq files were deposited in the Sequence Read Archives (SRA) of the National Center for Biotechnology Information (NCBI) under the accession numbers Biosamples SAMN17606697, SAMN17606698, SAMN17606699, SAMN17606700, SAMN17606701, and SAMN17606702, regarding the non-infected, *T. equi*-infected profiles, respectively, of Bioproject PRJNA695199. The *de novo* assembly fasta file has been deposited at Transcriptome Shotgun Assembly (TSA) database under the accession number GIZL00000000. However, a total of 46,484 contigs were excluded from the fasta file due to its size (lower than 200 nucleotides) or matches with UniVec vector sequences protected with copyrights. The estimation of transcript abundance was performed using the RNA-Seq by Expectation-Maximization (RSEM) method [62]. The open reading frames (ORFs) present in the transcripts were predicted using the TransDecoder tool version 5.5.0 [63]. The coding potential was also assessed by the tool formerly described [64], which applies the taxa "6941 *Rhipicephalus microplus*" from NCBI. This tool can distinguish coding from non-coding transcripts using the transcripts’ specific properties, creating mathematical models from well-known coding and non-coding sequences, and assigning a score to each transcript. The assembled transcripts were identified through their similarity with proteins deposited in the UniProt database through the OmicsBox platform [65]. The annotation of each sequence was made based on the Basic Local Alignment Research Tool (BLAST) results, comparing the transcript to all the organisms belonging to the taxon "Ixodidae" and "Apicomplexa" in the UniProt database (accessed on 19 September 2019). The completeness of the *R. microplus* salivary gland transcriptome was evaluated using BUSCO (Benchmarking Universal Single-Copy Orthologs) by comparing the transcriptome against a set of highly conserved single-copy orthologs of the known ancestral Arthropoda proteins (arthropoda_odb10, creation date: 20 November 2019, number of species: 90, number of BUSCOs: 1013) [66,67]. The functional annotation of each sequence was obtained by comparing the transcripts with the Gene Ontology database (http://geneontology.org/) [68], the EggNOG mapper [69], and the InterPro tool v.5.0 [70]. Data related to family, repetitions, domains, and protein sites, such as protein families (Pfam) and proteins functionality (PANTHER), in addition to the identification of enzymes, were reviewed, then the results were combined and recorded for each transcript. Annotation can be defined as the process of discovering essential components of the genome, mainly genes, and their products, adding to them the analyses and the interpretations necessary to extract their biological importance. The functions were organized into biological, structural, or metabolic processes and were analyzed in graphs from the notes. The comparison between the generated profiles was performed according to the most present GO category level 2.

The differential expression analysis was performed using the edgeR package from R software [71]. Transcripts with *p*-values < 0.05 were considered differentially expressed.

### 4.6. Validation of RNA-seq Data

#### 4.6.1. Target Transcripts

Thirty differentially expressed transcripts in the large-scale sequencing were selected to be confirmed by real-time PCR assays. The primers were designed using Primer Express 3.0 software (Thermo Fisher Scientific, Wilmington, DE, USA) (Supplementary Table S4). The characteristics of the primers were tested using Oligo Explorer 1.2 software at http://www.genelink.com/tools/gl-oe.asp. The primers’ specificity was tested using the BLAST tool (https://www.ncbi.nlm.nih.gov/tools/primer-blast/index.cgi). Each primer pair’s efficiency was assessed through standard curves constructed with six-fold serial dilutions of *R. microplus* cDNA following the instructions described in the Minimum Information for Publication of Quantitative Real-Time (MiQE) guidelines [72]. The endogenous control primers used were already described [73]. The stability of the endogenous controls β-tubulin, β-actin, and GAPDH genes was assessed in the current study with the samples.

#### 4.6.2. Gene Expression Measured by qPCR Assays

A second experiment was performed following the same method described in the previous section to confirm the first experiment’s results. From each condition, a pool of 15 *R. microplus* SG was used to extract total RNA using the TRIzol^®^ Plus RNA Purification System (Thermo Fisher Scientific, Wilmington, DE, USA). The quality of RNA was analyzed by electrophoresis on a 1% agarose gel. The High-Capacity cDNA Synthesis Kit (Thermo Fisher Scientific, Wilmington, DE, USA) was used to convert RNA to cDNA. The qPCR assays were performed using the Step One Plus Real-Time PCR System (Applied Biosystems, Thermo Fisher). Designed primers were submitted to optimization, and PCR efficiency and amplification specificity were determined by applying a cDNA serial dilution curve. Subsequently, each sample was tested in triplicate using customized primers (Thermo Fisher Scientific, Wilmington, DE, USA) at 0.4 µM sense and antisense, 1x Power SYBER Green kit (Thermo Fisher Scientific, Wilmington, DE, USA), and 60 ng/µL cDNA in a total volume of 12 µL. The cycling conditions were as follows: an initial cycle of denaturation at 95 °C for 10 min, followed by 40 cycles of 95 °C for 15 s and 60 °C for 1 min. Fluorescence readings were taken at 60 °C after each cycle, and a dissociation curve (60–95 °C) was performed. Negative standard controls were prepared with molecular grade water. The Spearman correlation test was applied to verify the correlation between the mRNA levels by RNA-Seq and qPCR techniques.

## 5. Conclusions

The present study generates knowledge on the poorly studied tick cell–*Theileria* interplay by exploring the sialotranscriptomes of uninfected and *Theileria equi* infected *Rhipicephalus microplus* ticks. The RNA-seq strategy employed provided insight into the tick cellular response to infection and the *Theileria* molecular mechanisms during the parasite sporogony phase within the tick host cell. The identification of several *T. equi* transcripts related to the invasion mechanism suggests that the parasite uses similar approaches to the ones described on the parasite–vertebrate host cell stage during tick cell invasion. Additionally, results highlight the possibility of parasite manipulation on an epigenetic level, driving the tick cell host to react in a specific manner. Although progress is visible in this initial study, further investigation targeting specific parasite stages could expand the understanding of tick–parasite interactions and transcriptional adjustments that likely enable the parasite to persist in the tick’s SGs.

On the tick side, a robust translational induction was observed as a response to infection with a very high number of differential expressed genes recorded. Such a fact advocates the prospect of a short co-evolution history between these organisms. Stress response was also highly affected by the infection, and an atypical imbalance of transcripts related to blood-feeding and mitigation of host defenses was observed, adding to the prior concept. Several molecules with potential impact in tick–pathogen interactions were pinpointed, paving the way for further studies, such as functional analyses, which can assist the discovery of new anti-tick vaccine targets as well as the identification of pharmacologically active proteins.

## Figures and Tables

**Table 1:** Assembly statistic of *Rhipicephalus microplus* sialotranscriptome.

Total Trinity transcripts	295,406
% GC	46.21
Contig ExN10	8044
Contig ExN20	5968
Contig ExN30	4649
Contig ExN50	2721
Median contig length	340
Average contig	961.12
Total assembled bases	283,930,859

## Data Availability

The data presented in this study are openly available in the SRA database, reference number BioProject: PRJNA695199, BioSample accessions: SAMN17606697, SAMN17606698, SAMN17606699, SAMN17606700, SAMN17606701, and SAMN17606702. The *de novo* assembly fasta file has been deposited at Transcriptome Shotgun Assembly (TSA) database under the accession number GIZL00000000.

## Supplementary Materials

The following are available online at https://www.mdpi.com/2076-0817/10/2/167/s1, Table S1: Identification of all transcripts and their respective functional annotations, Table S2: Differentially expressed transcripts, down-regulated in the Theileria equi-infected experimental condition, Table S3: Differentially expressed transcripts, up-regulated in the Theileria equi-infected experimental condition, Table S4: Primer sequences (5′ to 3′) for qPCR analysis.
